# Neurosarcoidosis: Current Perspectives on Diagnosis, Management, and Future Directions

**DOI:** 10.7759/cureus.69208

**Published:** 2024-09-11

**Authors:** Tanya Sinha, Sohaira Tahir, FNU Namal, FNU Vineesha, FNU Warsha, Zeeshan Ahmed, Syed Faqeer Hussain Bokhari, Muhammad Haris, Muhammad Mudasser Khan

**Affiliations:** 1 Internal Medicine, Tribhuvan University, Kathmandu, NPL; 2 Internal Medicine, Avicenna Medical College, Lahore, PAK; 3 Internal Medicine, Social Security Hospital, Faisalabad, PAK; 4 Internal Medicine, University Medical and Dental College, Faisalabad, PAK; 5 Internal Medicine, Liaquat National Hospital and Medical College, Karachi, PAK; 6 Internal Medicine, Jinnah Sindh Medical University, Karachi, PAK; 7 Medicine and Surgery, King Edward Medical University, Lahore, PAK; 8 Internal Medicine, King Edward Medical University, Lahore, PAK; 9 Internal Medicine, Rawalpindi Medical University, Rawalpindi, PAK

**Keywords:** biologic therapies, biomarkers, cerebrospinal fluid analysis, corticosteroids, cranial neuropathy, granulomatous inflammation, immunosuppression, multidisciplinary management, neuroimaging, neurosarcoidosis

## Abstract

Neurosarcoidosis, a manifestation of sarcoidosis affecting the central or peripheral nervous system, presents unique challenges in diagnosis and management. Neurosarcoidosis can manifest with a wide range of symptoms, including cranial neuropathies, seizures, meningitis, and cognitive impairments. The heterogeneity of presentations often leads to diagnostic delays and complications. Diagnosis relies on a combination of clinical features, neuroimaging, cerebrospinal fluid analysis, and evidence of systemic sarcoidosis. Recent advances in imaging techniques, including high-resolution MRI, positron emission tomography (PET) scans, and novel biomarkers, have improved diagnostic accuracy. However, distinguishing neurosarcoidosis from mimicking conditions such as multiple sclerosis remains challenging. Treatment typically begins with corticosteroids, often requiring long-term immunosuppression. Second-line agents such as methotrexate and mycophenolate mofetil are frequently used as steroid-sparing options. Biologic therapies, particularly Tumor necrosis factor-alpha (TNF-α) inhibitors like infliximab, have shown promise in refractory cases. The long-term management of neurosarcoidosis necessitates a multidisciplinary approach with regular monitoring of disease activity and treatment response. Despite advancements, significant knowledge gaps persist in understanding the etiology, pathophysiology, and optimal management of neurosarcoidosis. Future research directions include identifying specific biomarkers, developing targeted therapies, and exploring novel imaging techniques. The rarity and heterogeneity of neurosarcoidosis underscore the importance of multicenter studies and international collaborations to advance our understanding and improve patient outcomes. Emerging technologies and innovative therapeutic approaches offer promising avenues for enhancing diagnosis and treatment in the coming years.

## Introduction and background

Sarcoidosis is a multisystem granulomatous disorder of unknown etiology, characterized by the formation of non-caseating granulomas in various organs and tissues. This chronic inflammatory disease can affect virtually any organ system, but it most commonly involves the lungs, lymph nodes, skin, and eyes. The clinical presentation of sarcoidosis is highly variable, ranging from asymptomatic cases discovered incidentally to severe multiorgan dysfunction [1]. Epidemiologically, sarcoidosis affects individuals worldwide, but its prevalence and clinical manifestations vary significantly across different ethnic groups and geographic regions [2]. Prevalence ranges from 1-5 per 100,000 in East Asia (South Korea, Taiwan, and Japan) to 140-160 per 100,000 in Sweden and Canada [3]. The incidence is highest in Sweden (11.5 per 100,000), compared to 8-11 per 100,000 in the U.S. and 6.8 per 100,000 in Canada, while East Asia reports lower rates (0.5-1.3 per 100,000) [3]. Variability in data sources and methodologies, including the duration of data coverage and whether only hospitalization data is used, affects these estimates. Race significantly influences sarcoidosis rates in the U.S., with non-Hispanic Blacks, particularly women, having the highest incidence and mortality rates. Factors such as systemic racism, environmental exposures, and healthcare access disparities contribute to this increased burden [3].

While pulmonary involvement is the hallmark of sarcoidosis, neurological manifestations, collectively termed neurosarcoidosis, represent a significant and often challenging aspect of the disease with significant mortality. Neurosarcoidosis is defined as the involvement of the central or peripheral nervous system by sarcoid granulomas. It occurs in approximately 5-10% of patients with systemic sarcoidosis, although postmortem studies suggest that subclinical neurological involvement may be present in up to 25% of cases [4].

The importance of addressing neurosarcoidosis separately from systemic sarcoidosis cannot be overstated. Neurological involvement in sarcoidosis presents unique diagnostic and therapeutic challenges for several reasons. First, the clinical presentations of neurosarcoidosis are remarkably diverse, mimicking various neurological disorders and making diagnosis difficult. Manifestations can range from cranial neuropathies and meningitis to mass lesions and cognitive impairment. This heterogeneity often leads to delayed diagnosis and treatment [5]. Second, neurosarcoidosis can occur in isolation, without evident systemic involvement, further complicating the diagnostic process. Isolated neurosarcoidosis, although rare, occurring in less than 1% of sarcoidosis cases, poses a significant diagnostic dilemma as it lacks the typical systemic clues that might prompt consideration of sarcoidosis [6]. Moreover, the diagnostic approach to neurosarcoidosis differs from that of systemic sarcoidosis. While serum biomarkers like angiotensin-converting enzyme (ACE) and imaging studies such as chest X-rays are valuable in diagnosing systemic sarcoidosis, their utility in neurosarcoidosis is limited [4]. Neuroimaging, cerebrospinal fluid (CSF) analysis, and sometimes brain biopsy play crucial roles in the diagnosis of neurosarcoidosis.

Treatment of neurosarcoidosis also presents unique challenges [7]. The blood-brain barrier limits the penetration of many conventional sarcoidosis treatments, necessitating different therapeutic approaches. Additionally, the potential for severe and irreversible neurological deficits underscores the importance of prompt and effective treatment. Given these considerations, a comprehensive understanding of neurosarcoidosis is essential for clinicians across various specialties, including neurology, pulmonology, and rheumatology. This review aims to provide current perspectives on neurosarcoidosis, focusing on its varied clinical presentations, diagnostic approaches, and management strategies. By addressing the specific challenges associated with neurosarcoidosis, we hope to contribute to improved recognition, timely diagnosis, and optimal management of this complex manifestation of sarcoidosis.

## Review

Clinical presentation

Neurosarcoidosis is characterized by a remarkably diverse array of neurological manifestations, reflecting the potential involvement of any part of the nervous system. This heterogeneity in clinical presentation often poses significant diagnostic challenges for clinicians. Cranial neuropathies are among the most common manifestations of neurosarcoidosis, occurring in approximately 50-70% of cases [8]. The facial nerve (cranial nerve VII) is most frequently affected, resulting in facial palsy that may be unilateral or bilateral. Other commonly involved cranial nerves include the optic nerve (II), vestibulocochlear nerve (VIII), and the lower cranial nerves (IX-XII). Optic nerve involvement can lead to vision loss, while vestibulocochlear nerve dysfunction may cause hearing loss or vestibular symptoms [9,10].

Seizures represent another significant neurological manifestation, occurring in about 7-22% of patients with neurosarcoidosis [8]. These can be of various types, including generalized tonic-clonic seizures, focal seizures, or complex partial seizures. The underlying pathology often involves cortical or subcortical granulomatous lesions. Meningeal involvement is observed in approximately 40% of cases, presenting as acute or chronic meningitis [11,12]. Neurosarcoidosis often presents as pachymeningitits which should be differentiated from other forms of meningitis [13]. Patients may experience headaches, neck stiffness, and altered mental status. In some cases, meningeal involvement can lead to hydrocephalus due to obstruction of CSF flow [14]. Spinal cord involvement, although less common, can result in myelopathy with sensory disturbances, weakness, and bladder or bowel dysfunction [15]. This can mimic other causes of myelopathy, such as multiple sclerosis (MS) or spinal cord tumors.

Neuromuscular manifestations include peripheral neuropathy and myopathy. Peripheral neuropathy can present as mononeuropathy, mononeuritis multiplex, or polyneuropathy. Myopathy may cause muscle weakness and atrophy [16]. Neuroendocrine dysfunction, particularly involving the hypothalamic-pituitary axis, can occur in neurosarcoidosis. This may lead to diabetes insipidus, hyperprolactinemia, or other endocrine abnormalities [17,18]. Cognitive impairment and psychiatric symptoms, including depression and psychosis, have been reported in some cases of neurosarcoidosis, although their exact prevalence is unclear [19].

The clinical presentation of neurosarcoidosis is highly variable, not only in terms of the specific neurological systems affected but also in the course of the disease. Some patients may present with acute, severe symptoms, while others may have a more indolent course with gradually progressive neurological deficits (Table 1).

**Table 1:** Summary of the prevalence of various neurosarcoidosis manifestations. SIADH: syndrome of inappropriate antidiuretic hormone. *One individual patient can have one or more neurosarcoidosis manifestation(s). Adapted with permission from Voortman et al. [7]

Neurosarcoidosis manifestation*	Prevalence	Comments
Cranial nerve palsy	31-55%	Facial and optic nerves are the most commonly affected; uni or bilateral involvement
Chronic aseptic meningitis	16-37%	Subacute or chronic lymphocytic meningitis; dural involvement including pachymeningitis, dural mass mimicking meningioma
Spinal cord disease/myelitis	18-23%	Subpial intramedullary lesions, typically longitudinally extensive; myelitis predilection cervicothoracic
Cerebral parenchymal disease	21%	Small cortical or periventricular white matter lesions; mimicking multiple sclerosis or micro-ischemic lesions, larger solitary aggregates of granulomas can masquerade as neoplasms
Neuroendocrine (hypothalamo-pituitary) involvement	6-9%	Hormonal disturbances including hypothyroidism, hypogonadism, panhypopituitarism, SIADH
Hydrocephalus	9-10%	Communicating and noncommunicating hydrocephalus; combination with leptomeningeal enhancement along the skull base
Cerebral infarction	6%	Stroke can be because of in situ thrombosis, compression of a large vessel by a granulomatous mass, sinovenous thrombosis, and intracerebral hemorrhage.
Peripheral nervous system	17%	Large fiber involvement: most commonly axonal distal sensorimotor polyneuropathy or asymmetric polyradiculoneuropathy (nonlength dependent distribution)

Differential diagnosis

The diverse and often nonspecific nature of neurosarcoidosis symptoms necessitates a broad differential diagnosis. Several neurological conditions can mimic neurosarcoidosis, complicating the diagnostic process. MS is perhaps the most common condition confused with neurosarcoidosis. Both can present with white matter lesions on MRI and can affect multiple neurological systems. However, MS typically has a relapsing-remitting course and distinctive MRI features that can help differentiate it from neurosarcoidosis [20]. Other inflammatory or infectious conditions that can mimic neurosarcoidosis include neuromyelitis optica, acute disseminated encephalomyelitis, CNS vasculitis, and chronic meningitis caused by tuberculosis or fungal infections. Neoplastic processes, particularly primary CNS lymphoma or metastatic disease, can sometimes present with neurological symptoms and imaging findings similar to neurosarcoidosis [21].

The diagnostic criteria for neurosarcoidosis, as proposed by Zajicek et al. and later modified by Marangoni et al., categorize the diagnosis as definite, probable, or possible based on the combination of clinical presentation, imaging findings, and histological evidence [22,23]. However, applying these criteria in clinical practice can be challenging (Table 2). The gold standard for diagnosing neurosarcoidosis is demonstrating non-caseating granulomas in neural tissue [24]. However, neural biopsy is often not feasible due to the risks involved. Therefore, diagnosis often relies on a combination of clinical features, neuroimaging, CSF analysis, and evidence of systemic sarcoidosis [25]. Distinguishing neurosarcoidosis from its mimics often requires a multidisciplinary approach involving neurologists, radiologists, and sometimes neurosurgeons.

**Table 2:** Zajicek and Marangoni-modified criteria for neurosarcoidosis. ACE: angiotensin-converting enzyme; Ga Scan: gallium scan; BAL: bronchoalveolar lavage; HRCT: high-resolution computer tomography; NS: neurosarcoidosis

	Zajicek criteria [22]	Marangoni-modified criteria [23]
Certain NS	Positive nervous system histology	Positive nervous system histology
Probable NS	Signs of inflammation in CNS, positive histology for a systemic lesion, or a positive Kveim-Siltzbach test and/or positive results for at least two of the following tests: Ga scan, serum ACE level, and chest radiology	Signs of inflammation in CNS, positive histology for a systemic lesion, and/or positive results for at least two of the following tests: Ga scan, chest HRCT, BAL with a CD4: CD8 ratio >3.5 and a CD4: CD8 ratio >5 in the CSF
Possible NS	Absence of histological confirmation and ruling out other inflammatory pathologies	Absence of histological confirmation and ruling out other inflammatory pathologies

The updated consensus diagnostic criteria for neuro-sarcoidosis (NS) were published in 2018, categorizing patients by diagnostic certainty into definite, probable, and possible NS [26]. This classification is based on pathological and clinical features, with an emphasis on clinical phenotype and biopsy confirmation (Table 3).

**Table 3:** Clinical criteria for a diagnosis of neurosarcoidosis (2018 Neurosarcoidosis Consortium Consensus). NCS: nerve conduction study Adapted with permission from Bradshaw et al. [4]

Diagnosis	Criteria
Definite	(1) The clinical presentation and diagnostic evaluation suggest neurosarcoidosis, as defined by the clinical manifestations and MRI, CSF, and/or EMG/NCS findings typical of granulomatous inflammation of the nervous system after rigorous exclusion of other causes. (2) The nervous system pathology is consistent with neurosarcoidosis. Type a: Extraneural sarcoidosis is evident. Type b: No extraneural sarcoidosis is evident (isolated CNS sarcoidosis)
Probable	(1) The clinical presentation and diagnostic evaluation suggest neurosarcoidosis, as defined by the clinical manifestations and MRI, CSF, and/or EMG/NCS findings typical of granulomatous inflammation of the nervous system after rigorous exclusion of other causes. (2) There is pathologic confirmation of systemic granulomatous disease consistent with sarcoidosis.
Possible	(1) The clinical presentation and diagnostic evaluation suggest neurosarcoidosis, as defined by the clinical manifestations and MRI, CSF, and/or EMG/NCS findings typical of granulomatous inflammation of the nervous system and after rigorous exclusion of other causes. (2) No pathologic confirmation of granulomatous disease.

Advances in diagnostic techniques

Neuroimaging plays a crucial role in the diagnosis and management of neurosarcoidosis. MRI remains the gold standard for neuroimaging in suspected neurosarcoidosis due to its superior soft tissue resolution and ability to detect subtle abnormalities. MRI findings in neurosarcoidosis are diverse and can include leptomeningeal enhancement, parenchymal lesions, and cranial nerve involvement. Leptomeningeal enhancement, seen in up to 40% of cases, is often described as thin and linear, although nodular patterns can occur [27]. Parenchymal lesions typically appear as T2-hyperintense areas with variable enhancement patterns. These lesions can be solitary or multiple and may mimic other conditions such as MS or CNS lymphoma [4]. Recent advances in MRI techniques have improved the detection and characterization of neurosarcoidosis lesions. Diffusion-weighted imaging (DWI) can help differentiate between sarcoid granulomas and malignant lesions, with sarcoid lesions typically showing less restricted diffusion [28,29]. Susceptibility-weighted imaging (SWI) can detect microhemorrhages associated with granulomatous inflammation, providing additional diagnostic information [30]. Computed tomography (CT) plays a complementary role to MRI, particularly in detecting calcifications and bony involvement. CT is also useful in guiding biopsies of accessible lesions. Positron emission tomography (PET), particularly with 18F-fluorodeoxyglucose (FDG), has emerged as a valuable tool in the diagnosis and management of neurosarcoidosis [31,32]. FDG-PET can detect areas of active inflammation, which appear as hypermetabolic regions. This technique is particularly useful in identifying systemic sarcoidosis involvement and potential biopsy sites [7]. Moreover, FDG-PET can help monitor treatment response, with a decrease in FDG uptake correlating with clinical improvement.

CSF analysis is an essential component of the diagnostic workup for neurosarcoidosis. Common findings include mild to moderate pleocytosis (usually lymphocytic), elevated protein levels, and decreased glucose concentration [33]. CSF-ACE levels are elevated in some patients with neurosarcoidosis, but the sensitivity and specificity of this test are limited. Bridel et al. demonstrated that mean CSF-ACE levels were not significantly different between neurosarcoidosis and non-neurosarcoidosis patients, and with a cut-off value of 2, the sensitivity and specificity of c-ACE were 66.7% and 67.3%, respectively [34]. Other CSF markers that may support the diagnosis of neurosarcoidosis include elevated levels of beta-2 microglobulin, lysozyme, and soluble interleukin-2 receptor (sIL-2R) [35,36]. However, these markers are not specific to neurosarcoidosis and should be interpreted in the context of clinical and imaging findings. Mangioris et al. have explored the utility of CSF cytokine profiles in diagnosing neurosarcoidosis [37]. IL-6, BAFF, CXCL9, CXCL10, CXCL13, GM-CSF, IFN-gamma, and TNF-alpha levels were significantly elevated in CSF from neurosarcoidosis patients compared to controls (p<0.0001), along with IL-2 (p=0.0002), IL-8/CXCL8 (p=0.0008), IL-10 (p=0.0005), and IL-13 (p=0.0018).

While there is no single definitive biomarker for neurosarcoidosis, several blood and CSF markers can support the diagnosis and monitor disease activity. Soluble interleukin-2 receptor (sIL-2R) has emerged as a promising biomarker for sarcoidosis activity. Elevated serum sIL-2R levels have been reported to have higher sensitivity than ACE for detecting active sarcoidosis, including neurosarcoidosis. In a study by Eurelings et al., sIL-2R levels showed 88% sensitivity and 85% specificity for detecting sarcoidosis, outperforming ACE, which had 62% sensitivity and 76% specificity, as confirmed by receiver-operating characteristic (ROC) analysis (p<0.0001) [38]. However, sIL-2R is not specific to sarcoidosis and can be elevated in other inflammatory conditions. Chitotriosidase, an enzyme produced by activated macrophages, has shown potential as a biomarker for sarcoidosis [39]. Elevated serum chitotriosidase levels have been reported in sarcoidosis patients, including those with CNS involvement, and may correlate with disease activity. These markers may provide additional information about disease activity and treatment response, but their clinical utility in neurosarcoidosis is still being evaluated.

Therapeutic approaches

The management of neurosarcoidosis remains challenging due to the heterogeneity of clinical presentations and the lack of large-scale randomized controlled trials. Current treatment strategies are largely based on expert opinion, case series, and extrapolation from the management of systemic sarcoidosis (Table 4).

**Table 4:** Medications commonly used in the treatment of neurosarcoidosis. GI: gastrointestinal; TB: tuberculosis; HBV: hepatitis B virus; CBC: complete blood count; LFT: liver function tests; TPMT: thiopurine methyltransferase; PCP: *Pneumocystis* pneumonia Adapted with permission from Bradshaw et al. [4]

Medication	Dose	Adverse effects	Monitoring	Comments
Glucocorticoids		Numerous including psychosis, osteoporosis, Cushing syndrome, hypertension, diabetes mellitus, gastric ulcers, glaucoma, and cataracts	Blood pressure and blood glucose	Give with calcium, gastric protection and consider PCP prophylaxis for doses ≥20 mg/d prednisone for ≥3 mo; monitor BP and lipids.
Prednisone	0.25–1 mg/kg/d PO			
Methylprednisolone	1,000 mg/d x 3–5 d IV			
Immunosuppressant agent				
Azathioprine	Up to 2-2.5 mg/kg PO daily	Anemia, neutropenia, hepatitis, and rarely lymphoma	CBC and LFTs	TPMT activity/genotype; close safety laboratory monitoring
Methotrexate	10–25 mg weekly PO or SQ	Cytopenias, hepatitis, pneumonitis, mucositis, teratogenicity, and GI upset	CBC, LFTs, and creatinine	Methotrexate dosing in this context is weekly and not daily. Give with folic acid 1 mg PO daily.
Mycophenolate mofetil	1–1.5 g PO BID	Anemia, GI upset, hepatitis, and colitis	CBC and LFTs	Teratogenicity warning; skin checks for malignancy
Tumor necrosis factor inhibitors			CBC	
Infliximab	3-7 mg/kg IV at weeks 0, 2, and 6 and then 3-7 mg/kg IV q4-8 wk	Infusion reactions, antidrug antibodies, malignancy, demyelination, hepatitis, and drug-induced lupus		Contraindicated in heart failure and multiple sclerosis; test for TB and HBV before treatment.
Adalimumab	40 mg SQ q2wk			Infliximab is often dosed with an oral immunosuppressant as above to reduce the risk of neutralizing antibodies and for synergy.

Corticosteroids form the cornerstone of initial therapy for neurosarcoidosis. They are typically the first-line treatment due to their potent anti-inflammatory effects [40]. The usual starting dose is prednisone 0.5-1 mg/kg/day, which is generally maintained for 4-6 weeks (guided by the clinical response to therapy) before initiating a slow taper. In cases of severe or life-threatening disease, such as hydrocephalus or spinal cord compression, high-dose intravenous methylprednisolone (1000 mg daily for 3-5 days) may be used initially, followed by oral prednisone. While corticosteroids are often effective in controlling acute symptoms, their long-term use is associated with significant side effects. These include osteoporosis, diabetes mellitus, hypertension, weight gain, and increased susceptibility to infections [41]. Therefore, steroid-sparing agents are often introduced early in the course of treatment to allow for steroid tapering.

Conventional immunosuppressants are frequently used as steroid-sparing agents or in patients who do not respond adequately to corticosteroids alone (as second-line agents). In a study by Lower et al., 80% of patients with neurosarcoidosis required treatment with a second- or third-line agent [12]. Methotrexate is one of the most commonly used second-line agents, typically started at 5-12.5 mg weekly and titrated up to 25 mg (2.5 to 5 mg every 1-2 weeks) as needed [42]. Folic acid supplementation is essential to mitigate side effects. Other frequently used immunosuppressants include mycophenolate mofetil (500-3000 mg daily in divided doses), azathioprine (2-3 mg/kg/day), and leflunomide (10-20 mg/day) [42]. These immunosuppressants each have their own side effect profiles that need to be carefully considered. Methotrexate can cause hepatotoxicity and bone marrow suppression, mycophenolate mofetil is associated with gastrointestinal side effects (switching to EC-mycophenolate sodium can be considered) and increased risk of infections.

The advent of biological therapies has opened new avenues for the treatment of neurosarcoidosis, particularly in cases refractory to conventional treatments. These targeted immunomodulatory treatments offer the potential for more specific interventions in the disease process. Tumor necrosis factor-alpha (TNF-α) inhibitors have shown promising results in the treatment of refractory neurosarcoidosis. Infliximab, a monoclonal antibody against TNF-α, has been the most extensively studied in this context [43,44]. Studies by Fritz et al., Aubart et al., and Gelfand et al. reported improvement or remission in 71%, 89%, and 77% of patients, respectively [44-46]. The typical dosing regimen is 3-5 mg/kg at weeks 0, 2, and 6, followed by maintenance infusions every 4-8 weeks. Adalimumab, another TNF-α inhibitor, has also shown efficacy in case series of neurosarcoidosis patients [47,48]. It offers the advantage of subcutaneous administration, typically 40 mg every 1-2 weeks. However, TNF-α inhibitors are associated with an increased risk of infections, particularly reactivation of latent tuberculosis, and careful screening is necessary before initiating treatment. Rituximab, a monoclonal antibody targeting CD20+ B cells, has emerged as a potential treatment option for refractory neurosarcoidosis. While the pathogenesis of sarcoidosis is primarily T-cell mediated, B cells may play a role in antigen presentation and cytokine production. Case reports and small series have described favorable outcomes with rituximab in neurosarcoidosis patients who failed other treatments [49-51]. The typical dosing regimen is 1000 mg given intravenously on days 1 and 15, with repeat courses considered based on clinical response.

The long-term management of neurosarcoidosis requires a multidisciplinary approach and careful monitoring of disease activity and progression. Regular clinical evaluations, coupled with neuroimaging and laboratory tests, are essential to assess treatment response and detect any signs of relapse. MRI plays a crucial role in monitoring disease activity. Serial MRI scans can help assess the response to treatment, with a reduction in enhancement and T2 signal abnormalities generally indicating improvement. However, the correlation between radiological findings and clinical status is not always straightforward, and clinical assessment remains paramount. Serum biomarkers such as ACE and soluble interleukin-2 receptor (sIL-2R) can be useful in monitoring disease activity, although their utility in neurosarcoidosis specifically is less well established. Some centers use serial measurements of these markers to guide treatment decisions, but they should always be interpreted in the context of clinical and radiological findings.

Strategies for long-term care in NS involve balancing the need for disease control with the risks of prolonged immunosuppression. The goal is to achieve the lowest effective dose of immunosuppressive medications that maintain disease remission, often involving a gradual tapering of corticosteroids while adjusting doses of steroid-sparing agents. Relapse prevention is crucial, though no standardized approach exists. Some strategies include prolonged maintenance therapy, with experts recommending continued low-dose immunosuppression even after apparent remission to prevent relapses. Regular follow-up for early detection and treatment of relapses should be individualized based on disease severity and treatment regimen. Patient education about the signs and symptoms of relapse facilitates early reporting and intervention. Addressing modifiable risk factors, such as managing general health and avoiding potential environmental triggers, may also be beneficial. The long-term prognosis of neurosarcoidosis is variable, depending on factors such as the extent of neurological involvement, timing of diagnosis and treatment initiation, and response to therapy, with some patients achieving long-term remission and others experiencing a chronic relapsing-remitting course requiring ongoing management.

Future directions and research opportunities

Despite significant advances in our understanding of neurosarcoidosis, several important gaps in knowledge persist, presenting opportunities for future research. One of the most pressing areas needing further investigation is the etiology of sarcoidosis. While various environmental and genetic factors have been implicated, the exact trigger for the disease remains elusive. Identifying specific etiological factors could pave the way for targeted prevention strategies and more effective treatments. The pathophysiological mechanisms underlying the neurological manifestations of sarcoidosis are not fully understood. Research is needed to elucidate the factors that determine central nervous system involvement and the variability in clinical presentations. This could include studies on the role of the blood-brain barrier, neuroinflammation, and potential neurodegenerative processes in neurosarcoidosis. There is a lack of validated biomarkers for disease activity and treatment response in neurosarcoidosis. While certain markers like ACE and sIL-2R are used, their specificity and sensitivity for neurological involvement are suboptimal. Identifying reliable biomarkers could significantly improve diagnosis, monitoring, and treatment decision-making. The optimal duration of treatment and long-term management strategies for neurosarcoidosis remain unclear. Research is needed to establish evidence-based guidelines for treatment tapering, maintenance therapy, and monitoring for relapses.

Emerging technologies and approaches offer exciting possibilities for improving the diagnosis and treatment of neurosarcoidosis. In the realm of diagnostics, advanced neuroimaging techniques show promise. Ultra-high field MRI (7 Tesla and above) could provide more detailed visualization of CNS lesions and potentially detect subtle abnormalities not visible on conventional MRI. Molecular imaging techniques, such as novel PET tracers targeting specific inflammatory markers, may offer more sensitive and specific detection of neurosarcoidosis activity. Artificial intelligence and machine learning algorithms applied to imaging and clinical data could potentially improve diagnostic accuracy and predict disease course. These technologies could assist in differentiating neurosarcoidosis from mimicking conditions and in identifying patients at higher risk of complications or treatment resistance. On the therapeutic front, several innovative approaches are being explored: targeted immunotherapies, such as JAK inhibitors and other small molecule immunomodulators, are showing promise in early studies; nanoparticle-based drug delivery systems could potentially improve the delivery of anti-inflammatory drugs across the blood-brain barrier, enhancing efficacy while reducing systemic side effects; cell-based therapies, including mesenchymal stem cells and regulatory T-cell therapies, are being investigated for their potential immunomodulatory effects in various inflammatory conditions, including sarcoidosis; and neuromodulation techniques, such as transcranial magnetic stimulation (TMS) or vagus nerve stimulation, could potentially be explored for managing certain neurological symptoms of sarcoidosis.

Given the rarity and heterogeneity of neurosarcoidosis, multicenter studies and large-scale registries are crucial for advancing our understanding and improving patient care. Collaborative research efforts can overcome the limitations of small, single-center studies by providing larger sample sizes, which are necessary for more robust statistical analyses and generalizable results. They also allow for the study of diverse patient populations, potentially uncovering geographic or ethnic variations in disease manifestations and treatment responses. Large-scale registries can provide valuable real-world data on the natural history of neurosarcoidosis, treatment patterns, and long-term outcomes. The Sarcoidosis Advanced Registry for Cures (SARC) in the United States and the European Sarcoidosis Registry are examples of such initiatives, but more focused neurosarcoidosis registries could provide even more targeted insights. Multicenter clinical trials are essential for evaluating new therapeutic approaches. Given the challenges in recruiting sufficient numbers of neurosarcoidosis patients at single centers, collaborative networks are crucial for conducting adequately powered studies. These networks can also facilitate the standardization of outcome measures and diagnostic criteria, enhancing the comparability of results across studies. International collaborations are particularly important in rare diseases like neurosarcoidosis. They can help in identifying genetic and environmental factors that may vary across populations and in developing globally applicable management guidelines. The World Association of Sarcoidosis and Other Granulomatous Disorders (WASOG) plays a crucial role in fostering such international collaborations.

## Conclusions

Neurosarcoidosis remains a challenging manifestation of sarcoidosis, requiring a high index of suspicion for timely diagnosis and management. The diverse clinical presentations and potential for isolated neural involvement necessitate a comprehensive diagnostic approach, integrating clinical, radiological, and laboratory findings. Advances in neuroimaging and biomarker research have improved diagnostic accuracy, but further validation is needed. Treatment strategies continue to evolve, with corticosteroids forming the cornerstone of initial therapy, often supplemented by steroid-sparing agents and biological therapies in refractory cases. The long-term management of neurosarcoidosis demands a delicate balance between disease control and minimizing treatment-related complications. Despite progress, significant knowledge gaps persist, particularly in understanding disease etiology, identifying reliable biomarkers, and optimizing long-term management strategies. Future research should focus on elucidating pathophysiological mechanisms, developing targeted therapies, and conducting large-scale collaborative studies. As our understanding grows, personalized treatment approaches may become feasible, potentially improving outcomes for patients with this complex neurological disorder.
